# Vasohibin-1 suppresses colon cancer

**DOI:** 10.18632/oncotarget.3493

**Published:** 2015-03-08

**Authors:** Shuai Liu, Bing Han, Qunyuan Zhang, Jie Dou, Fang Wang, Wenli Lin, Yuping Sun, Guangyong Peng

**Affiliations:** ^1^ Department of Oncology, Jinan Central Hospital, Affiliated to Shandong University, Jinan, P. R. China; ^2^ Department of Internal Medicine, Saint Louis University School of Medicine, Saint Louis, MO, USA; ^3^ Department of Obstetrics and Gynecology, Qilu Hospital of Shandong University, Jinan, P.R. China; ^4^ Department of Genetics, Washington University School of Medicine, Saint Louis, MO, USA

**Keywords:** VASH1, Colon cancer, Angiogenesis, Tumor suppressor, Metastasis

## Abstract

Vasohibin-1 (VASH1) is an endogenous angiogenesis inhibitor. However, the clinical relevance of VASH1 in colon cancer and its regulations on cancer angiogenesis and cancer cell biological characteristics are still unknown. Here we showed that stromal VASH1 levels were negatively correlated with tumor size, advanced clinical stage and distant metastases in colon cancer patients. Overexpression of VASH1 in colon cancer cells induced apoptosis and senescence, inhibiting cancer cell growth and colony formation *in vitro* and tumor growth *in vivo*. In addition, knockdown of VASH1 in cancer cells promoted cell growth, adhesion and migration *in vitro*, and enhanced tumorigenesis and metastasis *in vivo*.

## INTRODUCTION

Colorectal cancer (CRC) is a major cause of cancer death in the USA and worldwide. There are 100, 000 new cases of colon cancer and 50, 000 cases estimated deaths a year [1, 2]. Understanding the pathogenesis and regulatory processes during cancer development will provide novel strategies for colon cancer treatment. In addition to the genetically controlled development of tumor cells, tumor microenvironmental factors, such as tumor angiogenesis, are also critical in enabling tumor growth, progression and distant metastasis [3]. Angiogenesis is well defined as formation of neovessels and a key event involved in tumor oncogenesis. Homeostasis of tumor angiogenesis is controlled by a panel of angiogenesis stimulators and inhibitors expressed in tumor cells and stroma cells in the tumor microenvironment [3-7]. A better understanding of biological functions and regulations of those stimulators and inhibitors will provide novel targets for the effective antiangiogenic therapy against colon cancer and other cancers as well.

Vasohibin family has been recently identified as novel negative feedback regulators of angiogenesis [6, 8, 9]. There are two members in the vasohibin family, vasohibin-1 (VASH1) and its homologue vasohibin-2 (VASH2), but they may have distinct roles in angiogenesis regulation [10, 11]. VASH1 is selectively expressed on the endothelial cells, which is induced by angiogenesis stimulators such as vascular endothelial growth factor (VEGF) and fibroblast growth factor 2 (FGF-2) [6, 7]. VASH1 directly regulates endothelial cell fate and biological functions resulting in the angiogenesis inhibition [12-14]. VASH1 has also been confirmed as a critical angiogenesis regulator involved in tumor angiogenesis inhibition and prevention of tumor growth and metastasis in animal tumor models with lung carcinoma and hepatocellular carcinoma [15-18]. Importantly, recent studies retrospectively analyzing the relationships between clinicopathological features and tumor stroma VASH1 expression from cancer patients have further indicated that VASH1 is a novel prognosis molecular marker in various cancers, including in breast cancer [19], renal cell carcinoma [20, 21], lung cancer [22], upper urinary tract urothelial carcinoma [23], and hepatocellular carcinoma [24, 25]. However, little information is known about the role of VASH1 in the regulation of tumor angiogenesis, oncogenesis, and clinical outcomes of human colorectal cancer.

Although VASH1 was originally identified as endothelial cell-derived angiogenesis inhibitor, recent studies suggested that its expression is not restricted to the endothelial cells, but also in other types of cells including in cancer cells [18, 26, 27]. However, the functional role of VASH1 in directly regulating cancer cell biological characteristics is still under investigation. Especially, whether cancer cell-derived VASH1 can influence tumorigenesis and metastasis has not been fully characterized.

In our current efforts to explore the functional role of VASH1 in the pathogenesis of human colon cancer, we performed immunohistochemical staining of VASH1 in colon cancer tissues and paired paracancerous normal tissues from patients with different stages of primary colon cancer, and retrospectively analyzed the correlations between the cancer stroma VASH1 levels with tumor stages, metastases, prognostic factors and clinical outcome of patients. Furthermore, we also analyzed the VASH1 expression in colon cancer cells and determined its regulations on cancer cell fate and biological functions. We observed that patients with lower stroma VASH1 expression levels had bigger tumor sizes, advanced clinical stages, as well as increased other organ metastases. Importantly, using the loss-of-function and gain-of-function strategies, we found that VASH1 expression in tumor cells was critical for cell growth, adhesion and migration *in vitro*, and controlled tumorigenesis and metastasis *in vivo* in animal models. These data clearly suggest that Vasohibin-1 may function as a tumor suppressor in colon cancer that controls both cancer angiogenesis and cancer cell biological functions.

## RESULTS

### Expression of VASH1 in cancer stroma of colon cancer patients

**Figure 1:**
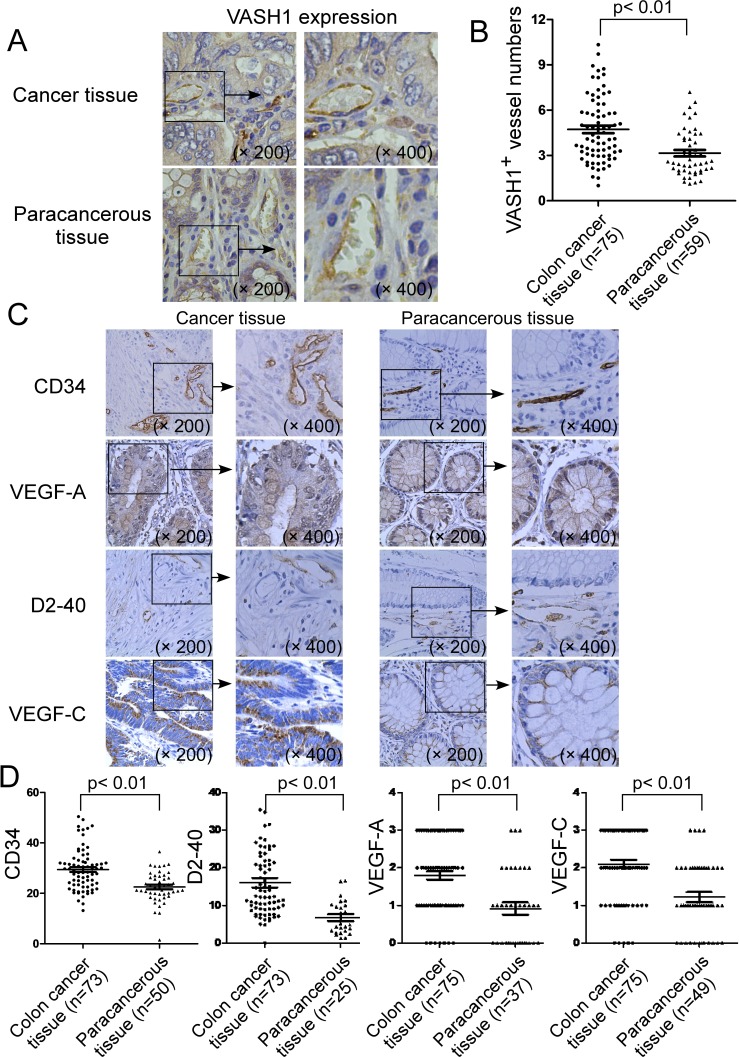
Expression of VASH1 in cancer stroma of colon cancer patients (A) & (B) Significantly increased VASH1 expression density in endothelial cells of blood vessels was detected in colon cancer stroma, compared with that expressed in paracancerous normal tissues. Numbers of VASH1^+^ vessels in 75 colon cancer tissues and 59 paracancerous normal tissues were detected and summarized using the immunohistochemical staining. (C) & (D) Expression levels of angiogenic molecules CD34 and VEGF-A, as well as lymphoangiogenenic molecules D2-40 and VEGF-C in colon cancer tissues (n=75) and paracancerous normal tissues (n=59) were determined using the immunohistochemical staining. Expression level of each dot shown in (B) and (D) is the average numbers (VASH1, CD34 and D2-40) or scores (VEGF-A and VEGF-C) per high field (400 x) in each tissue sample. The mean number of each molecule in each group is shown as a horizontal line. Significance was determined by unpaired (cancer tissue vs paracancerous tissue) T test.

Recent studies suggest that VASH1 is a novel angiogenic molecule that is critical for cancer angiogenesis and prognosis [19-25]. These novel findings prompted us to investigate the functional role of VASH1 in the pathogenesis of human colon cancer. We first performed immunohistochemical staining to detect VASH1 expression in 75 colon cancer tissues and 59 paracancerous normal tissues from cancer patients (Figure 1A & 1B). We found the prevalent expression of VASH1 in endothelial cells in both cancer stroma and paracancerous normal tissues (Figure 1A). However, in the paracancerous normal tissues, the numbers of VASH1^+^ vessels are very low (mean numbers of 3.1), whereas significantly increased numbers of VASH1 expression in vascular endothelial cells were detected in colon cancer stroma (mean numbers of 4.7) (Figure 1B). The result strongly suggested the activated angiogenesis in colon cancer patients. In addition, we investigated the expression levels of the other well-known angiogenic molecules CD34 and VEGF-A, as well as lymphoangiogenenic molecules D2-40 and VEGF-C in colon cancer tissues and paracancerous normal tissues (Figure 1C & 1D). CD34 expression was mainly localized in the cytoplasm and membrane of the blood endothelial cells, while D2-40 expression was observed in the cytoplasm and cellular membrane of lymph endothelial cells (Figure 1C). Furthermore, VEGF-A and VEGF-C were found expression in the cytoplasm both in cancer cells and in paracancerous normal tissues (Figure 1C). In addition, expression levels of CD34, D2-40, VEGF-A and VEGF-C in colon cancer tissues were significantly higher than those in paracancerous normal tissues (Figure 1D). Our results collectively suggest that both active angiogenesis and lymphoangiogenesis exist in colon cancer patients, and that VASH1 is prevalent in the cancer stroma of cancer tissues.

### Stroma VASH1 is an important cancer angiogenic molecule in human colon cancer

**Figure 2:**
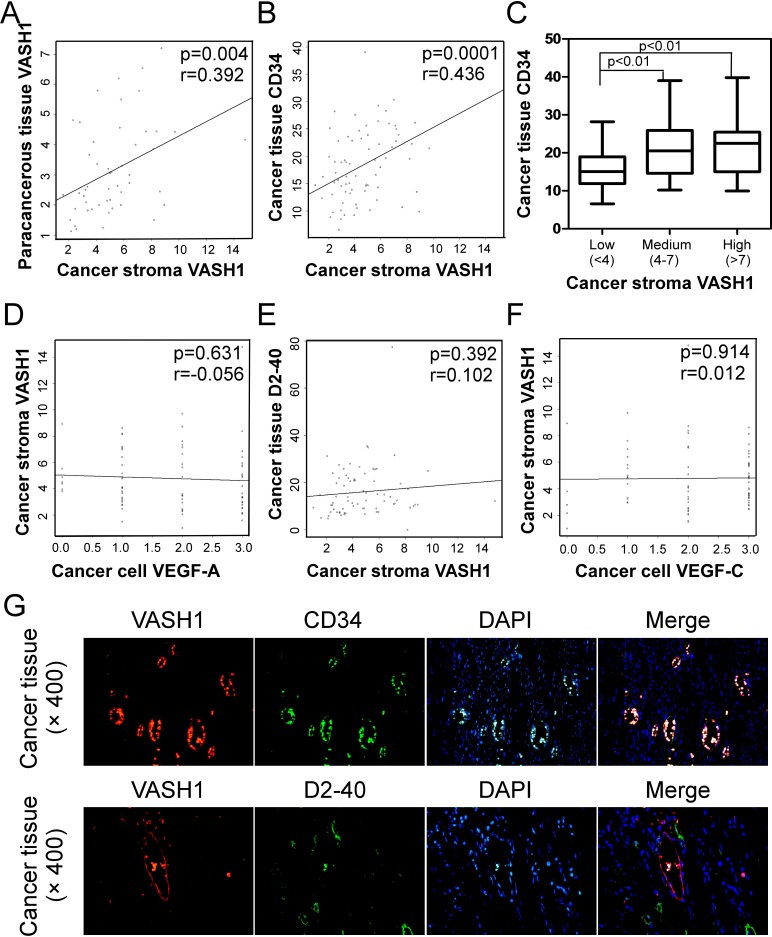
Correlations between cancer stroma VASH1 expression and levels of other angiogenic and lymphoangiogenenic molecules in colon cancer tissues (A) Scatter diagram showing a positive correlation between cancer stroma VASH1 and paracancerous tissue VASH1. (B) and (C) Scatter diagram (B) and box plot (C) analyses showing positive correlations between expression levels of cancer stroma VASH1 and cancer tissue CD34. The mean number of VASH1 in each group is shown as a horizontal line (in C). (D), (E) and (F) Scatter diagrams showing that there are no correlations between cancer stromal VASH1 expression and VEGF-A expression in cancer cells (D), and lymphoangiogenenic molecules D2-40 (E) and VEGF-C in cancer tissues (F). Expression levels of different molecules in colon cancer tissues (n=75) and paracancerous normal tissues (n=59) were immunohistochemically determined as described in Figure 1. (G) VASH1-exressing endothelial cells were also co-expressed with CD34, but not with D2-40, in the same blood vessels in cancer tissues. Immunofluoresence double staining with anti-VASH1 and anti-CD34, or anti-D2-40 antibodies in the same sections from colon cancer tissues was performed.

Given that high density of VASH1 expression in blood endothelial cells in cancer stroma, and that active angiogenesis and lymphoangiogenesis were observed in colon cancer tissues, we next determined whether cancer stroma VASH1 is associated with colon cancer lymphangiogenesis and angiogenesis. The correlations between cancer stroma VASH1 expression level and expressions of CD34, D2-40, VEGF-A, VEGF-C in cancer tissues were analyzed. We found that cancer stroma VASH1 was positively correlated with its expression in paracancerous normal tissues (Figure 2A). Furthermore, box plot and linear correlation analyses demonstrated that there was a significant correlation between stroma VASH1 and CD34, a key microvessel density (MVD) marker, in colon cancer tissues (Figure 2B and 2C). However, there were no correlations between cancer stroma VASH1 expression and VEGF-A expression in cancer cells, and lymphoangiogenenic molecules D2-40 (a lymphatic vessel density marker) and VEGF-C in cancer tissues (Figure 2D, 2E and 2F).

To further investigate the functional effect and correlation of VASH1 and CD34 involved in the active angiogenesis in colon cancer, we determined whether VASH1 expression was co-localized with CD34 in endothelial cells in cancer stroma. Immunofluoresence double staining with anti-VASH1 and anti-CD34, or anti-D2-40 antibodies in the same sections from colon cancer tissues was performed. As shown in Figure 2G, VASH1-expressing endothelial cells were also co-expressed with CD34 but not with D2-40 in the same vessels in cancer tissues. In addition, serial tissue sections with immunohistochemical staining analyses further confirmed that VASH1 and CD34 molecules were co-expressed in blood endothelial cells in colon cancer tissues (Supplemental Figure 1A). We then investigated the functional role of VASH1 as a critical inhibitor in angiogenesis using the *in vitro* HUVEC tube formation assay [28]. Human VASH1 has two isoforms of VASH1-A (the major VASH1 isoform) and VASH1-B (the alternative splicing isoform) [12]. Both VASH1-A and VASH1-B genes were transfected into human umbilical vein endothelical cells and their effects on the angiogenenic process were evaluated based on the numbers of branch points in the formation of endothelial tubules [28, 29]. As expected, HUVECs transfected with control vector rapidly adhered and formed the endothelial tubules. However, expression of VASH1-A and VASH1-B in HUVECs significantly attenuated angiogenic tube formation, further confirming their inhibitory effect on angiogenesis (Supplemental Figure 1B). Notably, this result is different from a previous study showing that only VASH1-B can inhibit migration and proliferation of endothelial cells [12]. Taken together, these data suggest that VASH1 is a critical antiangiogenic molecule rather than a marker for lymphoangiogenesis in colon cancer patients.

### Stroma VASH1 expression level is a significant prognostic factor in colon cancer patients

To investigate the clinical significance of VASH1 in colon cancer, the cancer clinicopathological factors of colon cancer patients were retrospectively analyzed relative to the stroma VASH1 expression levels. The median numbers of VASH1^+^ vessels in cancer stroma of colon cancer samples was used as a cutoff point to define the VASH1-high and VASH1-low groups. Fisher test was used to analyze the correlations between stroma VASH1 and those clinical factors. As shown in Table 1, stroma VASH1 expression levels (numbers of VASH1^+^ vessels) were strongly negatively correlated with tumor size (p=0.02), advanced clinical stage (p=0.006), and increased other organ metastases (p=0.003) in colon cancer patients. However, we didn't find any correlations between stroma VASH1 expression levels with other key clinicopathological factors, including tumor pathologic types, tumor differentiation stages and clinical outcomes of RFS and OS, which are different from the recent findings from the patients with other types of cancers [19-25]. Notably, our studies also demonstrated that VASH1 expression levels in normal paracancerous tissues were also negatively correlated with higher tumor sizes (Table 1). In addition, the correlations between expression levels of CD34, VEGF-A, D2-40, and VEGF-C with clinical factors were also analyzed. We only found significant positive correlations between VEGF-A expression levels with advanced clinical stages (p=0.007) and increased distant metastases (p=0.004) of colon cancer patients (Supplemental Table 1). These results collectively suggested that stroma VASH1 is an important negative regulatory factor in colon cancer tumorigenesis and progression.

**Table 1:** Correlations between tumor stroma VASH1, paracancerous tissue VASH1, and clinicopathologic characteristics in colon cancer patients

	Colon cancer stroma VASH1			Paracancerous tissue VASH1		
Parameters	<4.2	>=4.2	P	<2.6	>=2.6	P
**Gender**						
Female	12	15		9	9	
Male	25	23	0.632	16	16	1
**Age**						
<65	16	21		12	15	
>=65	21	17	0.358	13	10	0.571
**Pathologic types**						
Mucinous	9	6		5	4	
Tubular	28	32	0.399	20	21	1
**Tumor sizes**						
<20	13	24		10	18	
>=20	24	14	**0.021**	15	7	**0.0450**
**TNM stages**						
I	1	6		1	3	
II	15	22		14	16	
III	12	9		6	5	
IV	9	1	**0.006**	4	1	0.426
**Tumor differentiation**						
High	0	1		0	0	
Moderate	28	26		19	19	
Low	9	11	0.700	6	6	1
**Lymph node metastasis**						
No	22	29		18	19	
Yes	15	9	0.142	7	6	1
**Overall survival**						
Alive	9	11		6	7	
Death	10	8	0.745	5	6	1
**Disease-free survival**						
No	7	9		4	7	
Yes	2	2	1	2	0	0.192
**Distant metastasis**						
No	28	37		21	24	
Yes	9	1	**0.003**	4	1	0.189

Note: Evaluated by Fisher test.

### Expression of VASH1 in colon cancer cells

**Figure 3:**
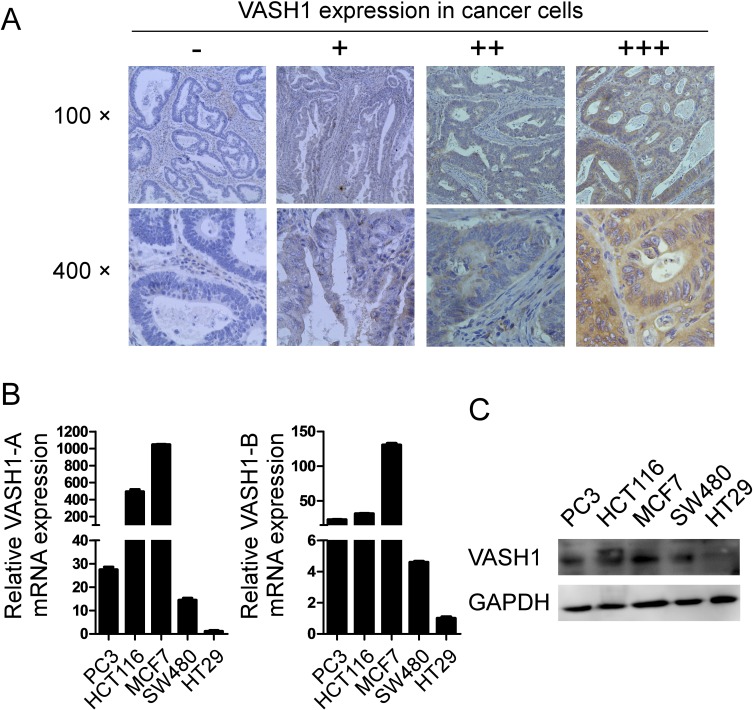
Expression of VASH1 in colon cancer cells (A) VASH1 expression was detected in the cytoplasm of colon cancer cells with varied expression densities using the immunohistochemical staining. VASH1 expression levels in cancer cells were classified into 4 grades according to its intensity: −, no expression; +, low expression with weak intensity; ++, moderate expression with moderate intensity; and +++, high expression with strong intensity. (B) Gene expression levels of VASH1-A and VASH1-B in different cancer cell lines, including colon cancer (HT29, HCT116 and SW480), prostate cancer (PC3), and breast cancer (MCF7), using Real-time PCR analyses. mRNA levels in each cancer cell line were normalized to the relative quantity of GAPDH expression, and then further compared to the expression level in HT29 cells (set as 1). Results shown in the histogram are mean ± SD from three independent experiments. (C) Protein expression levels of VASH1 in different cancer cell lines were determined using western blot analyses. GAPDH expression in cancer cell lysates was included as a control.

In addition to the prevalent expression of VASH1 in endothelial cells in cancer stroma, recent studies have shown that VASH1 can express in cancer cells [24, 30]. We thus determined whether VASH1 is also expressed in colon cancer cells. As expected, VASH1 expression was detected in the cytoplasm of colon cancer cells with varied expression densities (Figure 3A). Given that stroma VASH1 expression level is a significant prognostic factor in colon cancer development, we also retrospectively analyzed the correlations of VASH1 expression levels in cancer cells with the clinicopathological factors of colon cancer patients. Interestingly, we found that VASH1 expression levels in colon cancer cells were solely positively associated with the distant metastases (p=0.016), but were not correlated with the other factors or clinical outcomes (Supplemental Table 2), further suggesting that VASH1 is important for the dismal prognosis of colon cancer.

To further confirm VASH1 expression in cancer cells, we first determined the gene expression levels of VASH1-A and VASH1-B in different cancer cell lines, including colon, prostate, and breast cancers, using Real-time PCR analyses [12]. We found that all the tumor cell lines expressed both VASH1-A and VASH1-B with varied levels (Figure 3B). Among the three colon cancer cell lines, HT29 cell line is with lowest expression levels for both VASH1A and VASH1-B, while HCT116 cell line is with the highest expression for both of the VASH1 isoforms. VASH1 protein expression levels in different tumor cell lines were further confirmed using western blot analysis (Figure 3C). In addition, we determined the gene expression of VASH2, the other important VASH family member, in different cancer cell lines. Recent studies have shown that VASH2 also plays a critical role in tumor pathogenesis [31]. Our results showed that all the tumor cell lines also expressed VASH2 (Supplemental Figure 2). However, the VASH2 expression patterns in colon cancer cell lines are different from that of VASH1 expression. HT29 and SW480 cells were highly expressed VASH2 compared with that in HCT116 cells.

### VASH1 overexpression in colon cancer cells inhibits cancer cell growth, proliferation and colony formation

**Figure 4:**
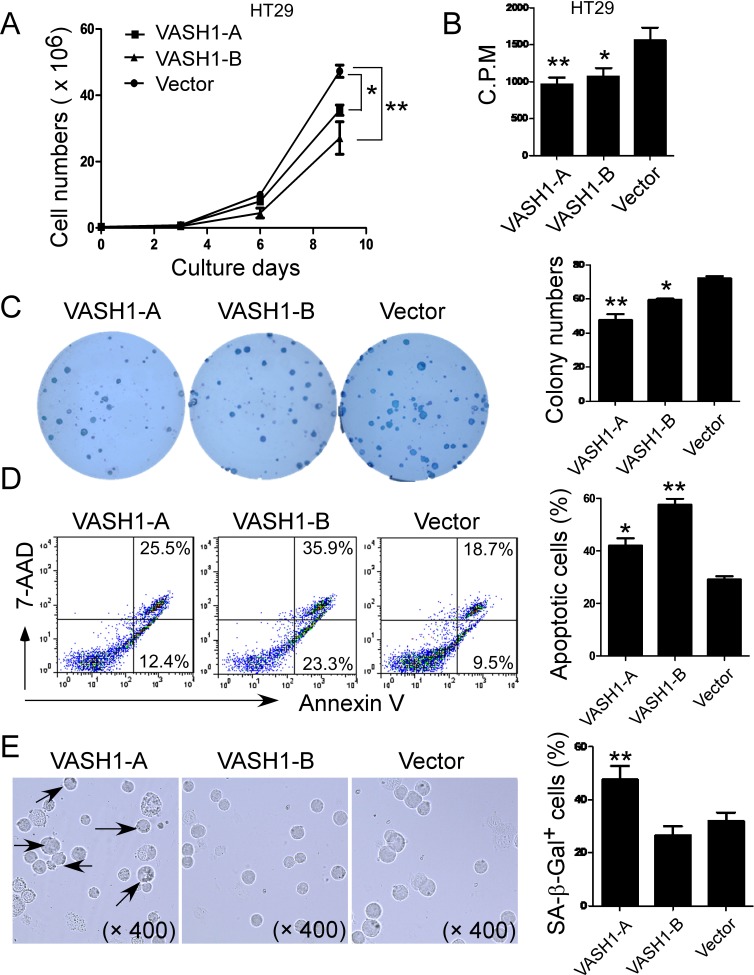
Overexpression of VASH1 in colon cancer HT29 cells significantly inhibits cancer cell growth, proliferation and colony formation (A) and (B) Transfection of both VASH1-A and VASH1-B in HT29 tumor cells significantly inhibited cell growth and proliferation. HT29 cells transfected with vector served as a negative control. Transfected HT29 cells were cultured at a started number of 3 × 10^5^/well in 6-well plates, or 5 × 10^3^/well in 96-well plates. The cell growth was evaluated at different time points using cell number counting (in A), and cell proliferation was determined using [3H]-thymidine assays (in B). (C) Transfection of both VASH1-A and VASH1-B dramatically decreased the numbers and sizes of tumor colonies in HT29 cells after 3 weeks of culture. Two hundred to five hundred per well of HT29 cells transfected with VASH1-A & B or control vector were seeded in 6-well plates and cell colonies counted after 3-4 weeks of culture. Results shown in the histogram are mean ± SD from three independent experiments. (D) and (E) VASH1-mediated inhibition of HT29 cell growth and proliferation was due to the induction of cell apoptosis and senescence. Significantly increased apoptotic cell populations were induced in HT29 cells after transfection with VASH1-B (in D). However, transfection with VASH1-A, but not VASH1-B in HT29 cells markedly induced SA-β-Gal positive cell populations in HT29 tumor cells (in E). Transfected HT29 tumor cells were cultured for additional 72 hours. Apoptosis in transfected tumor cells was analyzed after staining with PE-labeled Annexin V and 7-AAD (in D). Senescent cells were analyzed using the SA-β-Gal activity assay and the SA-β-Gal positive cells were identified with dark blue granules as indicated by the arrows (in E). Data in (A) to (E) are mean ± SD from three independent experiments with similar results. *p<0.05 and **p<0.01 compared with the vector control group.

Given that our studies showed VASH1 expression in both primary colon cancer cells and cancer cell lines, and that its intratumoral expression levels were associated with tumor metastases, we reasoned that VASH1 may directly influence cancer cell growth and metastasis capacity. To test these possibilities, we first selected HT29 cell line with low VASH1 expression for our gain-of-function studies. HT29 cells were transfected with VASH1-A or VASH1-B and tumor cell growth and proliferation determined using cell growth curve and [^3^H]-thymidine incorporation assays. The expression levels of VASH1-A and VASH1-B in HT29 cells after transfection were further confirmed by the Realtime-PCR analysis (Supplemental Figure 3). As shown in Figure 4A and 4B, transfection of both VASH1-A and VASH1-B in HT29 tumor cells significantly inhibited cell growth and proliferation. In addition, the colony-forming ability of the transfected HT29 tumor cells was also investigated. We observed that formed colonies of HT29 tumor cells were visible after 3 weeks of culture. Furthermore, the numbers and sizes of tumor colonies were significantly decreased in the HT29 cells transfected with VASH1-A and VASH1-B compared with those of control vector-transfected HT29 cells (Figure 4C).

We then dissected the potential mechanisms responsible for the inhibition of tumor growth and proliferation induced by VASH1 expression. Previous studies have shown that overexpression of VASH1-B in endothelial cells inhibited cell proliferation and DNA synthesis, as well as promoted cell apoptosis [12]. We reasoned that VASH1-mediated inhibition of HT29 tumor cells may also be due to the same mechanism as in endothelial cells. Apoptosis of HT29 tumor cells was analyzed at 48 hours post-transfection with VASH1-A and VASH1-B, using the 7-AAD and Annexin-V staining analyses. We observed significantly increased apoptotic cell populations in HT29 cells after transfection with VASH1-B (Figure 4D). Furthermore, transfection with VASH1-A also induced moderate levers of apoptosis in HT29 cells. Since senescent human cells have permanent growth arrest [32], we therefore determined whether senescence induction mechanism was also involved in the suppressed cell growth and proliferation mediated by overexpression of VASH1 in tumor cells. In addition to cell cycle arrest and morphologic characteristics, SA-β-Gal is the first biomarker used to identify senescent human cells [33-35]. As shown in Figure 4E, we found significantly increased SA-β-Gal positive cell populations in HT29 tumor cells after transfection with VASH1-A, indicating the induction of tumor cell senescence. In contrast, HT29 tumor cells transfected with VASH1-B and control vector did not induce SA-β-Gal expression. Our results collectively suggested that overexpression of VASH1 in colon cancer cells can induce both cell apoptosis and senescence, resulting in the inhibition of cancer cell growth and colony formation.

### VASH1 knockdown in colon cancer cells promotes cancer cell growth, adhesion and migration

**Figure 5:**
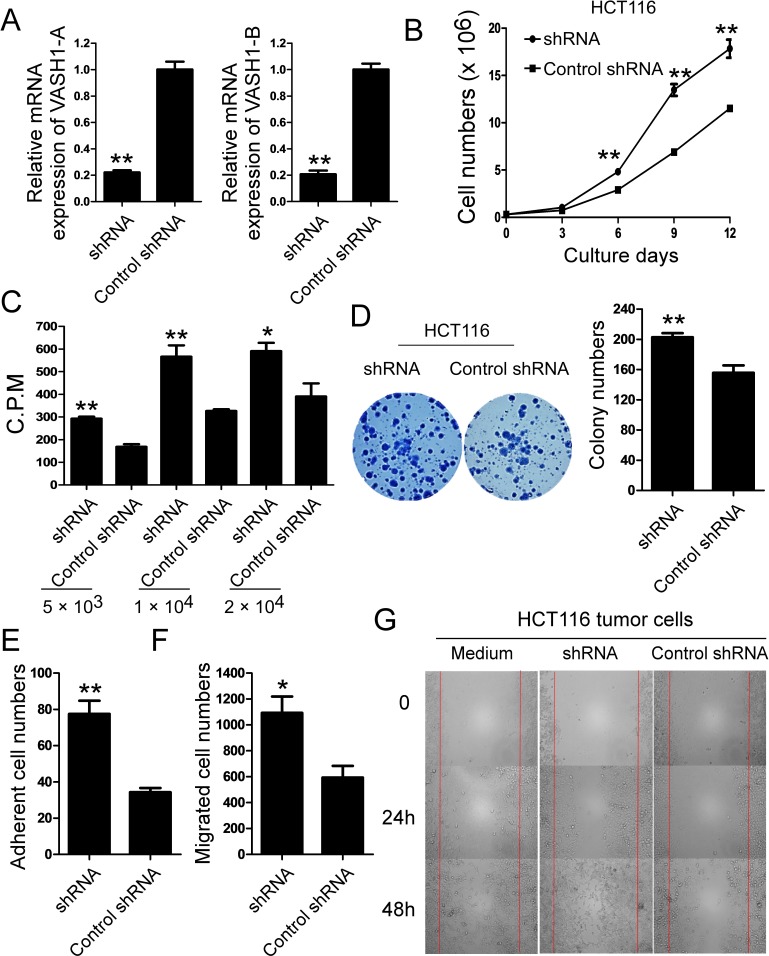
Knockdown of VASH1 in colon cancer HCT116 cells promotes cancer cell growth, adhesion and migration (A) The knockdown efficiency on VASH1-A and VASH1-B expression by the shRNA was determined in HCT116 tumor cells using Real-time PCR analyses. mRNA levels in each group were normalized to the relative quantity of GAPDH expression and compared against VASH1 expression level in control scramble shRNA group (set as 1). Results shown are mean ± SD from three independent experiments. **p<0.01 compared with the control scramble shRNA group. (B) and (C) Knockdown of VASH1 expression in HCT116 tumor cells dramatically promoted tumor cell growth and proliferation. HCT116 cells transfected with control shRNA served as a negative control. The cell growth of transfected HCT116 cells was evaluated at different time points using cell number counting (in B), and cell proliferation was determined using [3H]-thymidine assays (in C). **p<0.01 compared with the control scramble shRNA group. (D) Knockdown of VASH1 expression in HCT116 cells dramatically decreased the numbers and sizes of tumor colonies in colony formation assays. Results shown in the histogram are mean ± SD from three independent experiments. **p<0.01 compared with the control shRNA group. (E) Knockdown of VASH1 gene in HCT116 tumor cells markedly increased the adherent ability of tumor cells. The adhesion of transfected colon cancer cells was cultured in the fibronection-coated plates for 45 minutes. Adherent cells were counted and averaged in 10 fields at high (× 400) magnification with a microscope. **p<0.01 compared with the control shRNA group. (F) and (G) knockdown of VASH1 gene in HCT116 tumor cells significantly promoted the migration of tumor cells compared with the control shRNA-transfected tumor cells in the transwell migration assays (in F) and the wound closure assays (in G). *p<0.05 and **p<0.01 compared with the control shRNA group. Data in (A) to (G) are from three independent experiments with similar results.

To further confirm the functional role of VASH1 in regulating colon cancer cell growth, we also utilized the loss-of-function strategy to knockdown VASH1 gene with shRNA in VASH1 highly expressed HCT116 tumor cells and then determined its effect on tumor growth and proliferation. We selected a shRNA which can specifically target both VASH1-A and VASH1-B isoforms (Supplemental Figure 4A). The knockdown efficiency on VASH1 expression by the shRNA was further confirmed in both VASH1-transfected 293T cells and in HCT116 tumor cells, using western-blot and Real-time PCR analyses, respectively (Supplemental Figure 4B and 4C, and Figure 5A). As expected, silence of VASH1 expression in HCT116 tumor cells dramatically promoted tumor growth and increased cell proliferation (Figure 5B and 5C). Furthermore, the numbers and sizes of tumor cell colonies were also significantly increased in HCT116 cells after knockdown of VASH1 gene in a colony formation assay (Figure 5D). These results further suggested that VASH1 expression in colon cancer cells directly controlled cell growth and fate.

In addition to the tumor growth and colony formation, we next investigated whether VASH1 expression in tumor cells is critical for the capacity of cell adhesion and migration. We observed that knockdown of VASH1 in HCT116 tumor cells markedly increased the adherent ability of tumor cells in the fibronection-coated plates (Figure 5E). Furthermore, knockdown of VASH1 in HCT116 tumor cells significantly promoted the migration of tumor cells compared with the control shRNA-transfected tumor cells in a transwell migration assay (Figure 5F). The transmigration result was further confirmed in VASH1 shRNA-transfected HCT116 tumor cells using the wound closure assays (Figure 5G). These data collectively indicate that VASH1 in tumor cells is important for the regulation of tumor cell functions, including adhesion, migration and metastasis.

### VASH1 expression in tumor cells controls tumorigenesis and metastasis *in vivo*

**Figure 6:**
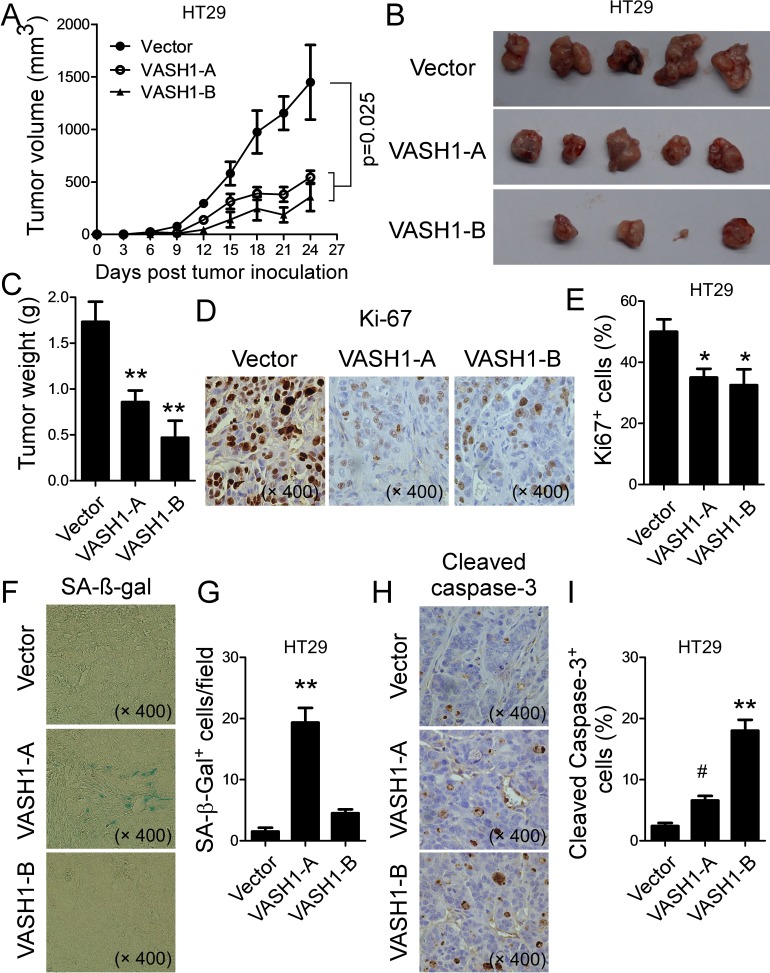
Overexpression of VASH1 in human colon cancer HT29 cells inhibited tumor growth and tumorigenesis *in vivo* (A) Overexpression of VASH1-A and VASH1-B in HT29 cells dramatically inhibited tumor growth in nude mice. (B) Representative image of the xenograft tumors shown are obtained from the 3 groups at the endpoint of the experiments (day 24). (C) The mean weights of xenograft tumors obtained from the 3 groups at the endpoint of the experiments (day 24). Human colon cancer HT29 cells (5 × 10^6^/mouse) transfected with VASH1-A, VASH1-B or control vector, were subcutaneously injected into nude mice (n=5). Tumor growth was evaluated (in A). At the end of experiments, tumors were isolated from the sacrificed mice and weighted (B). **p<0.01 compared with the control vector group (in B). (D) & (E) Transfection of VASH1-A or VASH1-B in HT29 cells significantly decreased Ki-67^+^ cell populations compared with vector-transfected group in the tumor tissues. Representative pictures for the expression of Ki-67 in tumor tissue sections determined using the immunohistochemical staining (in D). **p<0.01 compared with the control vector group. (F) & (G) Transfection of VASH1-A significantly increased SA-β-Gal positive cell populations in the tumor tissues. Representative pictures for the expression of SA-β-gal in tumor tissue sections determined using the SA-β-gal staining. **p<0.01 compared with the control vector group. (H) & (I) Transfection of VASH1-A and VASH1-B markedly increased Cleaved-Caspase 3^+^ cells in the tumor tissues. Representative pictures for the expression of Cleaved-Caspase 3 in tumor tissue sections determined using the immunohistochemical staining. ^#^p<0.05 and **p<0.01 compared with the control vector group. Results shown in (C), (E), (G) and (I) are mean ± SD from 5 mice per group.

These *in vitro* studies provided us important information regarding the importance of VASH1 in controlling colon cancer tumor cell growth and metastasis. We next performed complementary *in vivo* studies, using human colon cancer cells in humanized nude and Rag1^−/−^ immunodeficient mouse models, and explored whether VASH1 is critical for the tumorigenesis and metastasis of colon cancer *in vivo* [36, 37]. We first performed xenograft models to investigate whether overexpression of VASH1 in colon cancer cells can inhibit tumor growth and tumorigenesis. Human colon cancer HT29 cells transfected with VASH1-A, VASH1-B or control vector, were subcutaneously injected into nude mice. Tumor growth was evaluated. At the end of experiments, tumors were isolated from different groups of the sacrificed mice and weighted. HT29 tumor cells transfected with control vector grew progressively in nude mice. However, transfection of VASH1-A or VASH1-B in HT29 cells dramatically inhibited tumor growth (Figure 6A). Furthermore, tumor sizes collected from the VASH1-A or VASH1-B transfected HT29 groups on day 24 post inoculation were much smaller than those in the control vector-transfected group (Figure 6B). In addition, the average tumor weights obtained from the VASH1-A- and VASH1-B-transfected groups also showed much lower than that of control vector group (Figure 6C). Besides the tumor growth, we also verified the effects of VASH1 overexpression on tumor cell proliferation, apoptosis, senescence, and angiogenesis in tumor tissues. A large numbers of Ki-67^+^ cells were observed in HT29 tumor cells transfected with control vector. In contrast, transfection of VASH1-A or VASH1-B in HT29 cells significantly decreased Ki-67^+^ cell populations in the tumor tissues (Figure 6D and 6E). In addition, consistent to our in *vitro* observations (Figure 4), transfection of VASH1-A significantly induced tumor cell senescence evidencing by the increased SA-β-Gal positive cell populations (Figure 6F and 6G); and transfection of VASH1-B markedly increased the apoptotic cells (cleaved caspase 3^+^ cells) in the tumor tissues (Figure 6H and 6I). However, we did not find significantly inhibitory effects on tumor angiogenesis mediated by VASH1-A & B overexpression in tumor cells based on CD34^+^ blood vessel analyses in vessels in tumor tissues (Supplemental Figure 5). These results clearly suggest that VASH1 overexpression in colon cancer cells directly suppresses tumor growth and tumorigenesis, resulting from the induction of tumor cell apoptosis and senescence.

**Figure 7:**
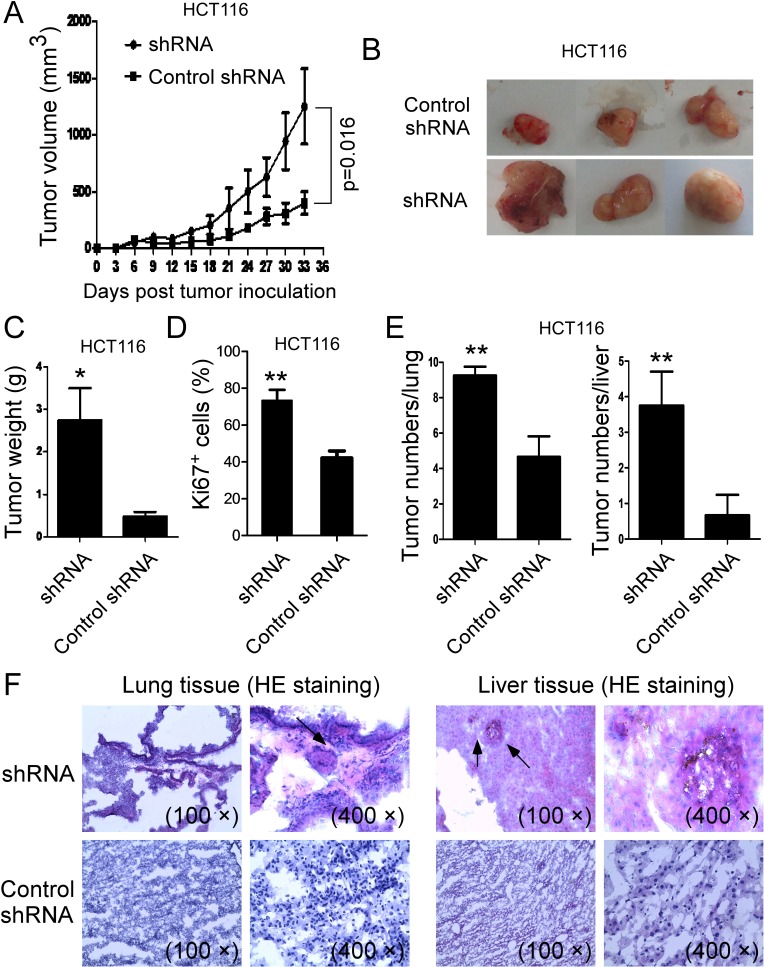
Knockdown of VASH1 in human colon cancer HCT116 cells promoted tumor tumorigenesis and metastasis *in vivo* (A) Knockdown of VASH1 in HCT116 cells dramatically promoted tumor growth in Rag1^−/−^ immunodeficient mice. (B) Representative image of the xenograft tumors shown are obtained from the two groups at the endpoint of the experiments (day 33). (C) The mean weights of xenograft tumors obtained from the two groups at the endpoint of the experiments (day 33). Human colon cancer HCT116 cells (4 × 10^6^/mouse) transfected with VASH1 shRNA or control shRNA, were subcutaneously injected into Rag1^−/−^ mice (n=5). Tumor growth was evaluated (in F). At the end of experiments, tumors were isolated from the sacrificed mice and weighted (G). *p<0.05 compared with the control shRNA group (G). (D) Knockdown of VASH1 in HCT116 cells significantly increased Ki-67^+^ cell populations compared with control shRNA group in the tumor tissues. Tumor tissue sections were determined for Ki-67 expression using the immunohistochemical staining. Results shown are mean ± SD from 5 mice per group. **p<0.01 compared with the control shRNA group. (E) Knockdown of VASH1 in HCT116 cells dramatically increased tumor macrometastatic numbers in lung and liver surfaces. Human colon cancer HCT116 cells (2 × 10^6^/mouse) transfected with VASH1-specific or control shRNA were injected tail intravenously into Rag1^−/−^ mice. Livers and lungs were harvested from the mice of different groups at 6 weeks post injection, and tumor metastases determined. Results shown are mean ± SD from 5 mice per group. **p<0.01 compared with the control shRNA group. (F) H & E staining on sections from embedded lung and liver tissues showed that high amount of tumor cells infiltrated into lungs and livers obtained from VASH1 shRNA treatment group, but not from the control shRNA treatment group. Tumor metastatic spots were indicated by the arrows.

We also performed a parallel xenograft model to investigate whether knockdown of VASH1 in colon cancer cells can promote tumor growth and tumorigenesis. Human colon cancer HCT116 cells transfected with VASH1 shRNA or control shRNA, were subcutaneously injected into Rag1^−/−^ mice. As shown in Figure 7A, knockdown of VASH1 in HCT116 cells dramatically promoted tumor growth, compared with the control shRNA–transfected HCT116 cells. In addition, tumor sizes collected from the VASH1 shRNA group on day 33 post inoculation were significantly larger than those in the control shRNA group (Figure 7B). Notably, the average tumor weights obtained from the VASH1 shRNA group also showed much higher than that of control shRNA group (Figure 7C). In addition, knockdown of VASH1 expression in HCT116 cells significantly increased Ki-67^+^ cell populations in the tumor tissues (Figure 7D). These data indicated that silence of VASH1 expression in tumor cells directly enhanced tumor growth and tumorigenesis *in vivo*.

We next investigated whether knockdown of VASH1 gene promoted tumor metastasis using our previously established adoptive transfer tumor models [36, 37]. Human colon cancer HCT116 cells transfected with VASH1-specific or control shRNA were injected tail intravenously into Rag1^−/−^ mice. Livers and lungs were harvested from the mice of different groups at 6 weeks post injection, and tumor metastases determined. As expected, knockdown of VASH1 in HCT116 cells dramatically increased tumor macrometastatic numbers both in lung and in liver surfaces (Figure 7E). These results are consistent with our findings in clinical association analyses in patient samples showing that VASH1 expression in stroma and cancer cells are highly related to the other organ metastases (Table 1 and Supplemental Table 2). Furthermore, we confirmed, using the H & E staining on sections from embedded liver and lung tissues, that high amount of tumor cells infiltrated into livers and lungs obtained from VASH1 shRNA treatment group, but not from the control shRNA treatment group (Figure 7F). Our studies collectively demonstrate that VASH1 is a tumor suppressor and plays a critical role in directing tumorigenesis and metastasis.

## DISCUSSION

Improved understanding of the molecular processes and regulations of tumor angiogenesis and oncogenesis will provide novel strategies for colon cancer treatment. Our current studies have identified that VASH1 functions as a significant tumor suppressor in human colon cancer. Based on the clinical sample analyses, we demonstrated that colon cancer stroma VASH1 is critical for cancer angiogenesis and its levels are strongly negatively correlated with tumor size, advanced clinical stage, and other organ metastases. Furthermore, cancer cell-derived VASH1 can directly regulate cell growth, adhesion and migration *in vitro*, as well as control tumorigenesis and metastasis *in vivo*. Our studies indicate that VASH1 could be not only a useful prognosis biomarker but also a novel therapeutic target for human colon cancer.

Tumor angiogenesis is a universal feature and also a key process for all cancers. VASH1 has been identified as a novel inhibitor involved in angiogenesis regulation [6-9]. Furthermore, VASH1 has also been found in cancer pathological conditions as a critical angiogenesis regulator involved in the tumor angiogenesis inhibition and prevention of tumor growth and metastasis in animal tumor models [15-18]. Based on the clinical sample analyses, our results strongly suggest that active angiogenesis and lymphoangiogenesis exist in colon cancer patients evidencing by the high expression levels of angiogenic molecules CD34 and VEGF-A, as well as lymphoangiogenenic molecules D2-40 and VEGF-C in colon cancer tissues [3, 5, 38-45]. We also confirmed that prevalent expression of VASH1 in endothelial cells in colon cancer stroma and paracancerous normal tissues, further suggesting the dynamic balance and regulation between angiogenic and antiangiogenic actions within the colon cancer tumor microenvironment. The major inducers for VASH1 expression in stroma endothelial cells are angiogenesis stimulators including VEGF-A [6, 7, 38]. Interestingly, we didn't observed a correlation between the cancer stroma VASH1 expression and cancer cell VEGF-A in colon cancer patients. However, significant correlation and co-expression between stromal VASH1 and microvessel density marker CD34 were found in colon cancer tissues. In addition, the *in vitro* HUVEC tube formation assay further indicated that both VASH1-A and VASH1-B genes could inhibit the angiogenenic process and the formation of endothelial tubules. These results indicated that VASH1 is an important cancer angiogenic molecule involved in the colon cancer antiangiogenesis.

Several recent studies have suggested that VASH1 could be a significant prognostic marker in various types of cancers [19-25]. In the current study, we retrospectively analyzed the correlations of stroma VASH1 expression levels with the cancer clinicopathological factors of colon cancer patients. Our studies demonstrated that stroma VASH1 expression levels were strongly negatively correlated with tumor size, advanced clinical stage, and other organ metastases in colon cancer patients. However, we didn't find correlations between stroma VASH1 expression levels with clinical outcomes of RFS and OS in colon cancer patients. Interestingly, our results are different from recent reports showing that VASH1 expression has significant positive correlation with pathological TNM stage, tumor stromal invasion, lymph node status and distant metastasis, and negative correlation with OS and RFS in colon cancer [27, 46]. In their studies, they provided mixed information and did not separate the analyses of clinical relevance with VASH1 expression in tumor cells or in endothelial cells. In addition, the difference between these two studies might be due to the small sample size and very limited follow-up information from cancer patients in our current study. Therefore, our future efforts should expand the colon cancer sample size to further confirm the functional role of VASH1 in the angiogenesis regulation and pathogenesis of colon cancer. Furthermore, it remains unclear how VASH1 performs antiangiogenic actions in colon cancer *in vivo*. Mechanistic studies using suitable animal models are needed to firmly establish the role of VASH1 in human colon cancer development.

VASH1 expression has been found in cancer cells including in colon cancer, but the mechanisms responsible for the regulation of cancer cell growth and biological characteristics or functions are unclear [18, 26, 27]. In addition, recent studies using recombinant adenovirus encoding VASH1 have shown that VASH1 inhibited tumor growth and metastasis *in vivo* [15, 16]. However, these studies only focused on how the administration of VASH1 regulated tumor angiogenesis and lymphangiogenesis, but didn't characterize the direct effects on tumor cells induced by VASH1 administration. In our current studies, we observed that VASH1 endogenously expressed both in primary cancer cells from colon cancer patients as well as in colon cancer cell lines. We further utilized the gain-of-function and loss-of-function strategies and discovered that cancer cell-derived VASH1 can directly control tumor cell fate and biological functions through the following actions. First, both VASH1-A and VASH1-B expression directly inhibit tumor cell growth and proliferation. Notably, the mechanisms responsible for VASH1-A and VASH1-B mediated tumor cell inhibition are different. We observed that VASH1-A expression in tumor cells significantly induced cell senescence [34, 35]. However, expression of VASH1-B dramatically induced apoptosis in colon cancer cells. These results are consistent with the findings in the endothelial cells showing that overexpression of VASH1-B inhibited DNA synthesis and induced apoptosis of endothelial cells [12]. In addition, VASH1 expression is critical for regulating cancer cell key biological functions, such as colony formation, adhesion and migration. Finally, VASH1 expression can control tumorigenesis and metastasis *in vivo* in human colon cancer models. Interestingly, our studies about the effect of VASH1-A on tumor cells are different from the observations in endothelial cells from other groups, showing that overexpression of VASH1-A does not affect endothelial cell growth and migration, but enhances cell ability for stress resistance to premature senescence and cell death [12, 14]. The difference may suggest that VASH1 has distinct functional roles varied among the cell types. In support of our assertion, a previous study has shown that overexpression of VASH1-A and B induced apoptosis in proliferating human fibroblasts, but did not affect cell growth of keratinocytes [12]. Notably, our retrospective analysis results showed that VASH1 expression levels in colon cancer cells were solely positively associated with the distant metastases, which is opposite to the results obtained from our *in vitro* and *in vivo* studies. The possibility may exist that VASH1 expressing in tumor cells is nonfunctional because of the mutation or methylation. In support of our prediction, a more recent study has suggested that enhancer of zeste homologue 2 (EZH2), a member of the polycomb group of genes (PcG), is overexpressed in human cancer tissues and can directly induce VASH1 methylation and promote tumor angiogenesis [4]. Furthermore, VASH1 mutations have been observed in colon, rectal and lung cancers [47-49]. Taken together, our studies identify that VASH1 functions as a significant tumor suppressor in human colon cancer that not only can inhibit cancer angiogenesis but also direct control cancer cell fate and biological functions. These studies could provide new insights relevant to the development of novel strategies using VASH1 as a useful target for human colon cancer treatment.

## MATERIALS AND METHODS

### Human samples and cell lines

Tumor samples of paraffin-embedded tissue sections and clinicopathological features were obtained from 75 colon cancer patients at Jinan Central Hospital affiliated to Shandong University from 2005 to 2008, who have undergone surgery and given informed consents for enrollment in a prospective tumor procurement protocol approved by the Shandong University Institutional Review Board. None of the patients had received any preoperative chemotherapy or irradiation before the operation.

Different types of tumor cell lines (breast, prostate and colon cancers), human umbilical vein endothelial cell (HUVEC) line, and HEK 293T cells were either purchased from the American Type Culture Collection (ATCC, Manassas, VA) or established in our laboratory. Human colon cancer cell lines SW480, HCT 29 and HCT116, as well as HEK 293T were maintained in DMEM medium containing 10% fetal calf serum (FCS). Other tumor cell lines PC3 and MCF7 were maintained in RPMI 1640 medium containing 10% FCS.

### Immunohistochemical staining and quantification method

The expression of VASH1, VEGF-A, CD34, VEGF-C and D2-40 (Podoplanin) in paraffin-embedded colon cancer tissue sections were determined using immunohistochemical staining with the following primary anti-human monoclonal antibodies: anti-VASH1(clone 4A3, Abnova Corporation, Taiwan, China), anti-VEGF-A (sc-152, Santa Cruz Biotechnology), anti-CD34 (ZA-0550, Zhongshan Jinqiao Co., Beijing, China), anti-VEGF-C (ZA-0266, Zhongshan Jinqiao Co) and anti-D2-40 (DAKO, Carpinteria, CA, USA), at diluted concentrations of 1: 1000, 1: 50, 1: 50, 1:100 and 1:200, respectively. The sections were stained for overnight at 4°C with primary antibodies and stained for 30 min at room temperature with secondary antibodies. Controls were performed by incubating slides with the isotype control antibody instead of primary antibodies, or a second antibody alone. The positive cells were counted and analyzed microscopically.

The stained sections were evaluated manually using a computerized image system composed of a Leica ICC50 camera system equipped on a Leica DM750 microscope (North Central Instruments, Minneapolis, MN). Photographs were obtained from 20 randomly selected areas within the tumor tissues of 10 cancer nest areas and 10 cancer stroma areas at a high-power magnification (400 ×). To analyze VASH1, CD34 and D2-40 expression in endothelial cells, ten fields (400 ×, magnification) of each tumor tissue section, including both cancer nest and stroma areas were counted, and the means of positive vessel numbers per field summed and reported. VASH1-positive vessels were counted in the “hot spot” that the highest number of the positive vessels for anti-CD34 was identified. VASH-1, VEGF-C and VEGF-A expression in tumor cells were evaluated semi-quantitatively based on immunostaining intensity and area extent. Each slide was given a score according to the intensity of cytoplasmic staining (0, negative; 1, weak; 2, moderate; 3, strong). The counting was performed by 2 independent investigators (S. Liu and B. Han) who had no previous knowledge of the patient clinical backgrounds, and the results were averaged.

### Indirect immunofluorescence staining

For indirect immunofluorescence staining of VASH1, CD34, and D2-40 in colon cancer tissues, paraffin-embedded sections were incubated with a mixture of mouse anti-human VASH1 (clone 4A3, Abnova Corporation, Taiwan, China) and rabbit anti-CD34 (ZA-0550, Zhongshanjinqiao, China), or rabbit anti-D2-40 (Cell Signaling Technology) primary antibodies. The sections were then incubated with a mixture of two secondary antibodies [Alexa Fluor 488-conjugated goat anti-rabbit IgG (H+L) and Alexa Fluor 594-conjugated goat anti-mouse IgG (H+L) (Proteintech Group, Inc, China)]. Specimens were then counterstained with 4′-6-Diamidino-2-phenylindole (DAPI) (Invitrogen).

### Gene constructs and cell transfection

The human VASH1A-p3xFLAG CMS-14 and VASH1B-p3xFLAG CMS-14 were generously gifted from Dr. Gerold Untergasser at the Innsbruck Medical University, Austria [12]. Human VASH-1 shRNA construct in pLKO.1-puro vector (NM_014909.2-1061s1c1) was purchased from Sigma. Scramble coding cDNA sequence for VASH1 shRNA was constructed with pLKO.1-puro vector and used as a control shRNA. Transfection of those constructs in tumor cells was performed using Lipofectamine 2000 (invitrogen) according to the manufacturer's instructions.

### HUVEC tube formation assay

Angiogenesis activity mediated by VASH1 expression was analyzed based on the human umbilical vein endothelial cell (HUVEC) tube formation assay, as previously described [28]. Briefly, HUVECs cultured in EGM with 5% fetal bovine serum and 1% endothelial cell growth supplement (ECGS) were transfected with VASH1-A, VASH1-B, or control vector. Transfected HUVECs were seeded at 2.5 × 10^4^ cells/well into matrigel-coated 48-well plates (BD Biosciences). The tube formation was observed after 6 hours of culture using an inverted phase contrast microscope and imaged by a Nikon digital camera, and then was quantified and summed by counting the numbers of branch points at a high-power magnification (200 ×).

### Reverse-transcription PCR analysis

Total RNA was extracted from tumor cells using Trizol reagent (Invitrogen), and cDNA was transcribed using a SuperScript II RT kit (Invitrogen), both according to manufacturers' instructions.

VASH1-A, VASH1-B and VASH2 mRNA expression levels were determined by reverse-transcription PCR using specific primers with the SYBR Green PCR Master Mix (Biosystems), and mRNA levels in each sample were normalized to the relative quantity of Glyceraldehyde-3-phosphate dehydrogenase (GAPDH) as previously described [36, 37]. The specific primers used are listed as follows: VASH1-A, F: 5′-CAAAGAGGCCCTGCCAATCA-3′ and R: 5′-TGGCGGAAGTAGTTCCCTGA-3′; VASH1-B, F: 5′-GACCTCTGACAGGGCTGATG-3′ and R: 5′-TCAACCTACCCCACCCTCAC-3′; and VASH2, F: 5′-GCTGGTCCTCAACGTCTCAA-3′ and R: 5′-CTGGGGGACAGGGATTTTCC-3′.

### Cell growth and functional proliferation assay

Colon cancer cell lines transfected with or without plasmids VASH1-p3xFLAG, VASH1 shRNA, or related controls, were cultured at a started number of 3 × 10^5^/well in 6-well plates. The cell growth was evaluated at different time points using cell number counting. In addition, cell proliferation of those transfected cells was determined using [^3^H]-thymidine assays as we previously described [36, 37]. In brief, different numbers of transfected tumor cells (5 × 10^3^, 1 × 10^4^ or 2 × 10^4^) were cultured in 96-well plates in cell assay medium containing 2% FCS. After 56 hours of culture, [^3^H]-thymidine was added at a final concentration of 1 μCi/well, followed by an additional 16 hours of culture. The incorporation of [^3^H]-thymidine was measured with a liquid scintillation counter.

### Western-blotting analysis

293T cells were transfected with VASH1A-p3xFLAG or VASH1B-p3xFLAG, combined with or without VASH1 shRNA, or scramble control shRNA for 48 hours. Transfected 293T cells were purified and then the lysates prepared for western blot analyses. An anti-Flag Monoclonal antibody (Clone M2, Sigma) was used in the western-blotting assays. To determine VASH1 protein expression in tumor cells, the lysates from different types of tumor cell lines were prepared for western blot analyses using an anti-VASH1 antibody (clone 4A3, Abnova Corporation).

### Cell apoptosis assay and senescence associated β-Galactosidase (SA−β-Gal) staining

Colon cancer cells transfected with VASH1-A, VASH1-B, or control vector were cultured for 72 hours. Apoptosis in transfected tumor cells were analyzed after staining with PE-labeled Annexin V and 7-AAD (BD Biosciences, San Diego, CA). All the stained cells were analyzed on a FACSCalibur (BD Bioscience) and the data were analyzed with FlowJo software (Tree Star, Ashland, OR). Furthermore, SA-β-Gal activity in tumor cells was detected as we previously described [34, 35]. Briefly, transfected tumor cells were cultured for 3 or 5 days and then were washed in PBS (pH 7.2), fixed in 3% formaldehyde, and followed to incubate overnight at 37°C with freshly prepared SA-β-Gal staining solution (1 mg/ml X-gal, 5 mM K_3_Fe[CN]_6_, 5 mM K_4_Fe[CN]_6_, 2 mM MgCl_2_ in PBS at pH 6.0). The stained cells were washed with H_2_O and examined SA-β-Gal expression with a microscope.

### Colony formation assay

Two hundred to five hundred per well of tumor cells transfected with VASH1-A & B, VASH1 specific shRNA, control vector or scramble shRNA, were seeded in 6-well plates for culture. Cell colonies were stained with Gimsa and counted after 3-4 weeks of culture.

### Wound closure and migration assays

HCT116 tumor cells were transfected with VASH1 shRNA or control shRNA. After 24 hours of transfection, the cells were wounded with a pipette tip across the cell monolayer. The scratches were photographed with a digital camera after additional 24 and 48 hours of culture. The closure was estimated as the wounded area relative to the initial area. For migration assays, 1 × 10^5^ transfected HCT116 tumor cells were put in the up-chambers with serum-free medium in the transwell assays (8 μm insert). The migrated cells in the lower chambers were collected and cell numbers counted after 12 hours of culture.

### Adhesion assay

The adhesion of transfected colon cancer cells was determined as previously described [50]. Flat bottom 96-well plates were coated with 50 μl per well of fibronectin (10 μg/ml, BD Biosciences) overnight at 4 °C and blocked with 2% BSA in PBS at 37 °C for 2h. The transfected tumor cells (2 × 10^5^/ml) suspended with basic medium without FBS and 10μl cells were seeded into each well and incubated for 45 minutes at 37 °C. After washing three times with PBS to remove non-adherent cells, the cells attached on the plates were fixed with 4% formaldehyde for 4 minutes and stained with 0.03% crystal violet 15 minutes. Adherent cells were counted and averaged in 10 fields at high (× 400) magnification with a microscope.

### *In vivo* tumorigenesis and metastasis studies

Rag1^−/−^ immunodeficient and nude mice (6-8 weeks) were purchased from The Jackson Laboratory (USA) and Laboratory Animal Center of the Academy of Military Medical Science (Beijing, China), respectively, and maintained in the institutional animal facility. All animal studies have been approved by the Institutional Animal Care Committee. For tumorigenesis studies, human colon cancer HT29 cells (5 × 10^6^/mouse) transfected with VASH1-A, VASH1-B or control vector, were subcutaneously injected into nude mice. In a parallel experiment, human colon cancer HCT116 cells (4 × 10^6^/mouse) transfected with VASH1 shRNA or control shRNA, were subcutaneously injected into Rag1^−/−^ mice. Five mice were included in each group. Tumor size was measured with calipers every 2-3 days. Tumor volume was calculated on the basis of the formula 1/2 (length^2^ × width). At the end of experiments, the mice were sacrificed and tumors were isolated and weighted. For evaluation of tumor cell proliferation, apoptosis, senescence, and angiogenesis in tissues, tumor tissues were prepared for paraffin-embedded sections (4~8 μm), and determined using the immunohistochemical staining or SA-β-gal staining, as described above. The primary antibodies include: anti-Ki-67 (Cell Signaling Technology), anti-cleaved Caspase-3 (#9664, Cell Signaling Technology), and anti-CD34 (ab81289, Abcam), at diluted concentrations of 1:50, 1:1000, and 1:200, respectively.

For tumor metastasis studies, transfected human colon cancer HCT116 cells (2 × 10^6^/mouse) were injected tail intravenously into Rag1^−/−^ mice. Five mice were included in each group. The mice were sacrificed and livers and lungs were harvested at 6 weeks post injection. Visible lung and liver surface macrometastatic white spots were counted using a dissecting microscope. For tissue morphology and metastasis evaluation, liver and lung tissues were embedded into OCT and prepared for cryostat sections (4~8 μm). Hematoxylin and eosin (H & E) staining were performed with sections from embedded samples.

### Statistical analysis

For human clinical sample analyses (in tables), given that there was no clinically defined cutoff points for the numbers of VASH1, VEGF-A, CD34, VEGF-C and D2-40 in the tumor tissues, the median expression of each molecule (4.2 for stroma VASH1; 2.6 for paracancerous tissue VASH1; 29 for CD34; and 12.4 for D2-40) in colon cancer tissues was used as a cutoff point to define the high and low expression groups (in tables). Fisher and Pearson's Chi-square tests were used to prospectively analyze the correlations between the expression levels of each marker and clinical features, including gender, age, pathologic types, clinical stages, tumor differentiation, lymph node metastases, other organs metastases, relapse-free survival (RFS), and overall survival (OS). OS was determined from the date of surgery to the date of death by any cause or to the date of the last follow-up. RFS was measured as the length of time from surgery to the date of relapse. Data processing and statistical analyses were performed using SAS 9.1 and R 2.13.0. Statistical significance was defined when alpha <0.05 (2-tailed). For other studies shown in figures, unless indicated otherwise, data are expressed as mean ± standard deviation (SD). The significance of difference between groups was determined by a two-tailed Student's *t*-test or the one-way analysis of variance (ANOVA).

## SUPPLEMENTARY MATERIALS FIGURES AND TABLES


