# Supplementary material for: Vasohibin-1 suppresses colon cancer
Source: Oncotarget. 2015 Mar 8;6(10):7880–98. doi: 10.18632/oncotarget.3493 (PMC4480723; doi:10.18632/oncotarget.3493)
Supplement: Supplementary file 1 [file oncotarget-06-7880-s001.pdf]

Vasohibin-1 suppresses colon cancer

Supplementary Material

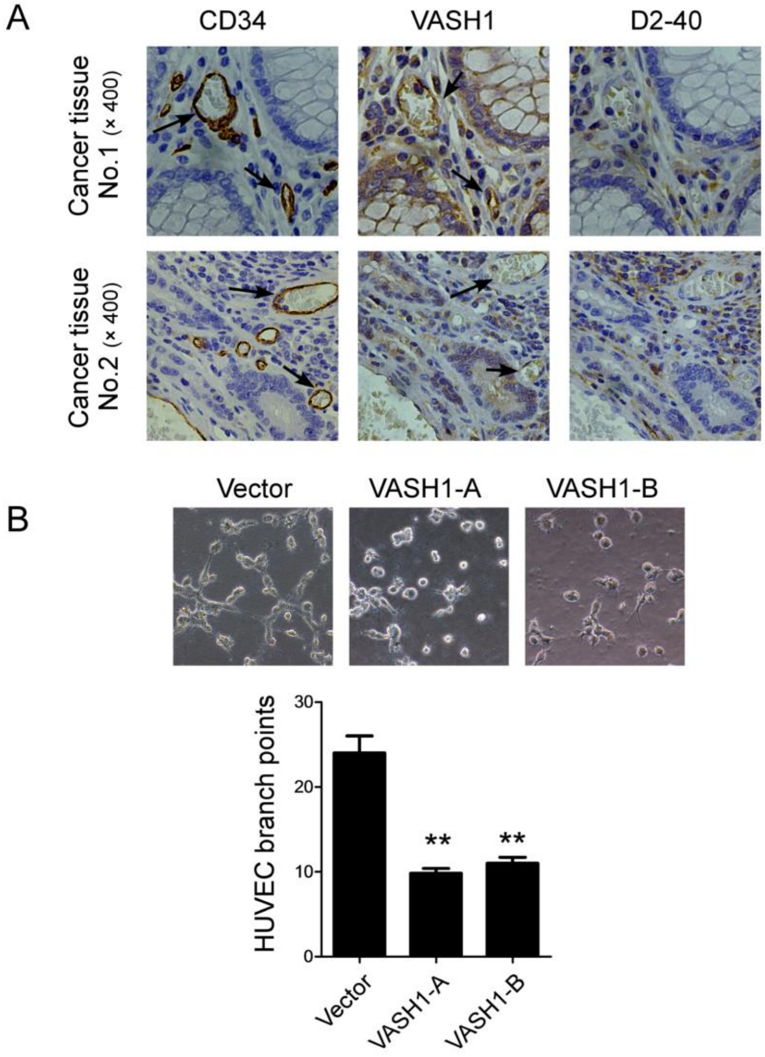

Supplemental Figure 1

VASH1 is a critical antiangiogenic molecule in colon cancer

(A) Co-expression of VASH1 with CD34, but not D2-40 was observed *in situ* in endothelial cells of the same blood vessels in serial colon tumor tissue sections. Immunohistochemical staining was performed to determine VASH1, CD34, and D2-40 expression in the serial sections from colon tumor tissues. **(B)** VASH1 functioned as an inhibitor in angiogenesis using the *in vitro* HUVEC tube

formation assay. Transfection of VASH1-A and VASH1-B in HUVECs significantly attenuated angiogenic tube formation of HUVECs evidencing by the decreased numbers of branch points in the formation of endothelial tubules. The upper pictures are representative images of the formed tubules from different groups. The lower histogram was the summary of quantitatively analyzed numbers of branch points from different groups at a high-power magnification (200 ×). Results shown in the histogram are mean ± SD from three independent experiments with similar results. **$p<0.01$ compared with the control vector group.

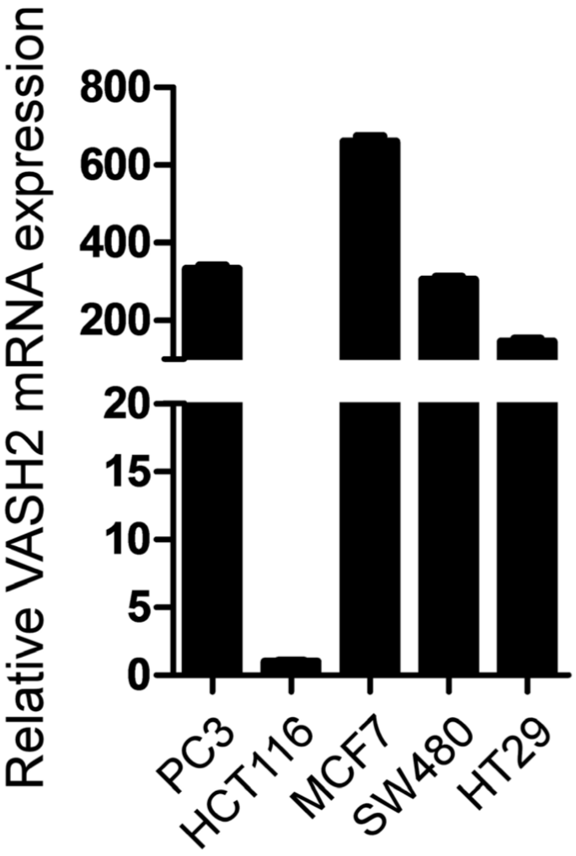

Supplemental Figure 2

VASH2 expression in different cancer cell lines.

Gene expression levels of VASH2 were determined in various cancer lines including colon cancer, prostate cancer, and breast cancer, using Real-time PCR analyses. mRNA levels in each cancer cell line were normalized to the relative quantity of GAPDH expression, and then further compared to the expression level in HCT116 cells (set as 1). Results shown in the histogram are mean ± SD from three independent experiments.

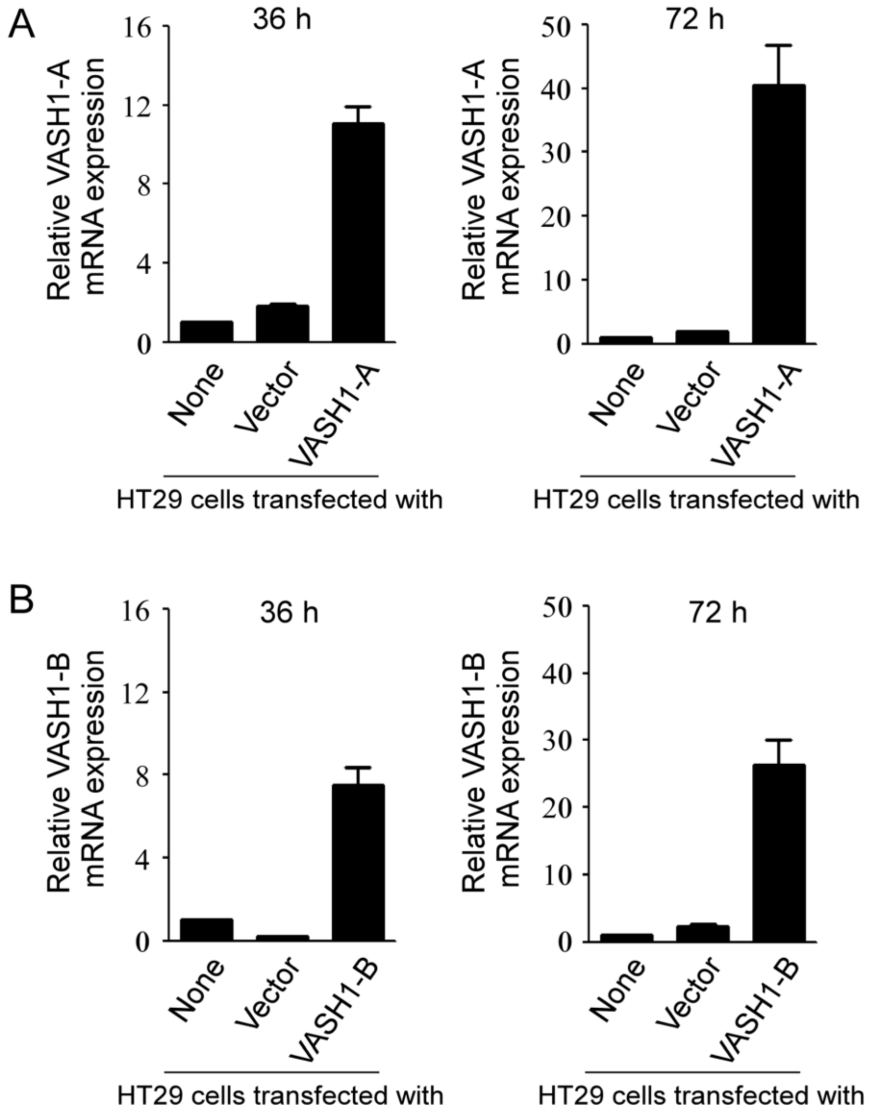

Supplemental Figure 3

Overexpression of VASH1-A and VASH1-B in HT29 cells.

mRNA expression levels of VASH1-A and VASH1-B in HT29 cells transfected with VASH1-A and VASH1-B genes were determined at 36 and 72 hours post transfection, using Real-time PCR analyses. mRNA levels in each cancer cell line were normalized to the relative quantity of GAPDH expression, and then further compared to the expression level in untransfected HT29 cells (set as 1). Results shown in the histogram are mean ± SD from three independent experiments.

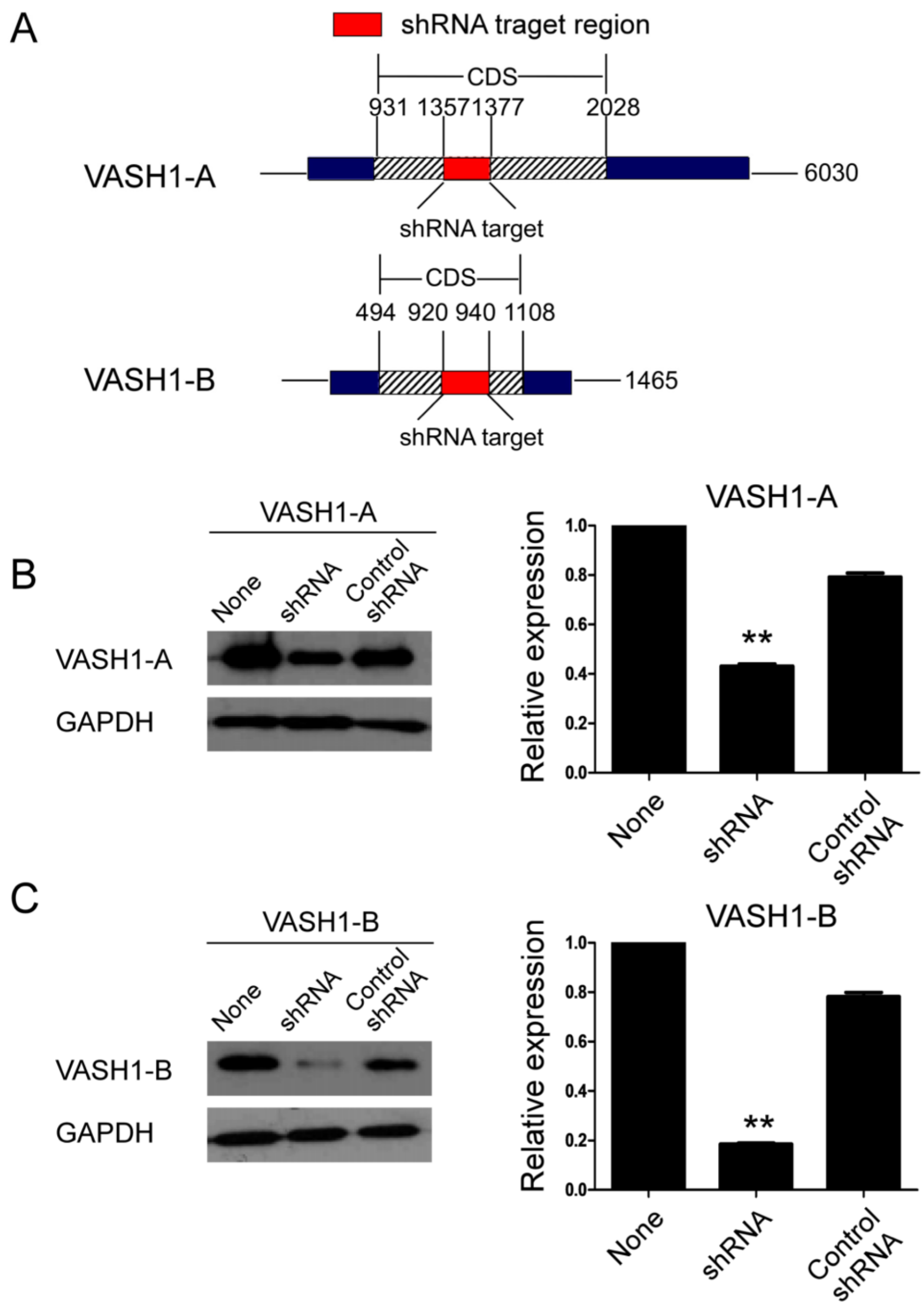

Supplemental Figure 4

The knockdown efficiency of shRNA on VASH1-A and VASH1-B expression.

(A) The gene map showing that VASH1 shRNA can target both VASH1-A and VASH1-B with gene regions of 1357 bp to 1377bp and 920 bp to 940 bp, respectively. **(B)** and **(C)** Transfection with VASH1 specific shRNA can significantly knockdown VASH1-A (in B) and VASH1-B (in C)

expression in 293T cells. 293T cells were transfected with VASH1A-p3xFLAG or VASH1B-p3xFLAG, combined with or without VASH1 shRNA, or scramble control shRNA for 48 hours. Transfected 293T cells were performed western blot analyses with an anti-Flag Monoclonal antibody. VASH1 expression levels shown in the right histogram were quantitatively analyzed and compared against VASH1 expression level in control group without transfection of shRNA (as 100%) with a densitometer. Results shown in the histogram are mean ± SD from three independent experiments. **$p<0.01$ compared with the control scramble shRNA group.

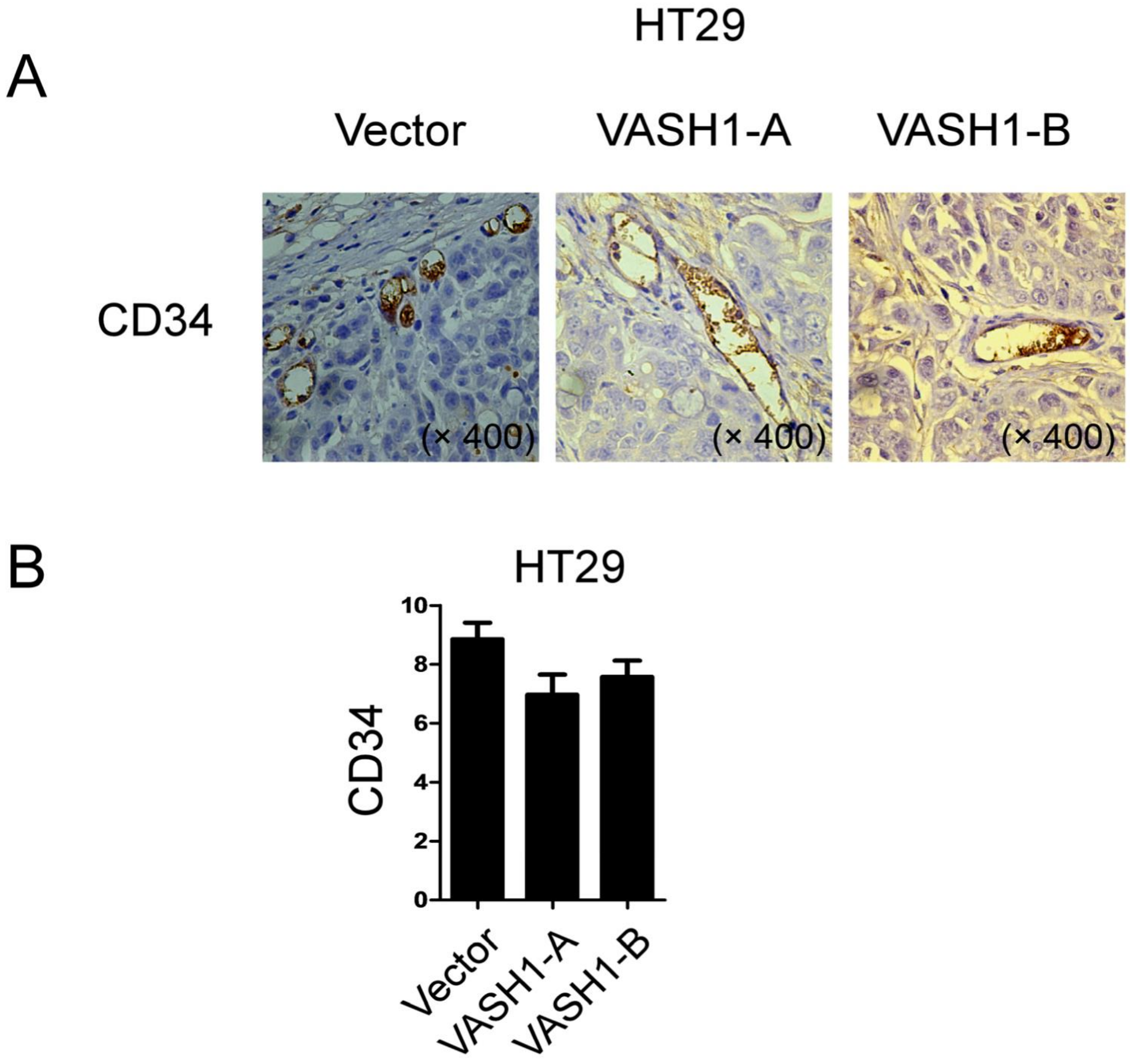

Supplemental Figure 5

Overexpression of VASH1 in human colon cancer HT29 cells did not significantly inhibit tumor angiogenesis *in vivo*.

(A) Representative pictures for the expression of CD34 in tumor tissue sections determined using the immunohistochemical staining. **(B)** Transfection of VASH1-A or VASH1-B in HT29 cells did not affect the numbers of $CD34^+$ blood vessels in tumor tissue sections using the immunohistochemical staining analyses. Results shown are mean ± SD from 5 mice per group.

Supplemental Table 1. Correlations between VEGF-A, VEGF-C, CD34, D2-40 and clinicopathologic characteristics in colon cancer patients.

	VEGF-A					VEGF-C					CD34			D2-40		
Parameter	0	1	2	3	P	0	1	2	3	P	<29	>=29	P	<12.4	>=12.4	P
Gender																
Female	2	6	9	10		2	6	8	11		15	12		15	11	
Male	5	19	10	14	0.367	3	8	16	21	0.931	21	25	0.475	21	26	0.334
Age																
<65	4	10	13	10		2	3	13	19		14	22		14	22	
>=65	3	15	6	14	0.226	3	11	11	13	0.108	22	15	0.065	22	15	0.103
Pathologic types																
Mucinous	2	4	4	5		2	4	5	4		7	7		5	8	
Tubular	5	21	15	19	0.826	3	10	19	28	0.291	29	30	1	31	29	0.542
Tumor size																
<20	2	14	11	10		2	10	11	14		16	20		19	18	
>=20	5	11	8	14	0.452	3	4	13	18	0.332	20	17	0.358	17	19	0.816
TNM stage																
I	3	1	2	1		1	3	1	2		5	2		3	4	
II	4	15	7	11		1	7	12	17		13	22		19	16	
III	0	9	7	5		1	2	7	11		11	10		8	13	
IV	0	0	3	7	**0.007**	2	2	4	2	0.267	7	3	0.178	6	4	0.614
Tumor differentiation																
High	0	1	0	0		0	1	0	0		1	0		0	1	
Moderate	4	20	12	18		4	9	15	26		24	29		26	28	
Low	3	4	7	6	0.425	1	4	9	6	0.324	11	8	0.608	10	8	0.689
Lymph node metastasis																
No	7	16	10	18		3	11	14	23		23	26		25	24	
Yes	0	9	9	6	0.109	2	3	10	9	0.565	13	11	0.625	11	13	0.804
Distant metastasis																
No	7	25	16	17		3	12	20	30		28	34		30	33	
Yes	0	0	3	7	**0.004**	2	2	4	2	0.113	8	3	0.531	6	4	0.344
Overall survival																
Alive	2	5	6	7		0	3	8	9		10	10		10	8	
Death	0	6	5	7	0.737	2	2	5	9	0.569	7	10	0.520	8	10	0.739
Disease-free survival																
No	2	4	6	4		0	3	5	8		7	9		7	7	
Yes	0	1	0	3	0.297	0	0	3	1	0.318	3	1	0.582	3	1	0.588

Note: Evaluated by Fisher test.

Supplemental Table 2. Correlations between colon cancer cell VASH1 and clinicopathologic characteristics in colon cancer patients.

	Colon cancer cell VASH1				
Parameter	0	1	2	3	P
Gender					
Female	5	8	10	4	
Male	7	22	12	7	0.559
Age					
<65	7	16	11	3	
>=65	5	14	11	8	0.460
Pathologic types					
Mucinous	0	7	4	4	
Tubular	12	23	18	7	0.144
Tumor size					
<20	6	11	15	5	
>=20	6	19	7	6	0.167
TNM stage					
I	1	2	4	0	
II	6	14	11	6	
III	3	11	6	1	
IV	2	3	1	4	0.325
Tumor differentiation					
high	0	1	0	0	
moderate	8	24	17	5	
low	4	5	5	6	0.174
Lymph node metastasis					
No	7	19	17	8	
Yes	5	11	5	3	0.621
Overall survival					
Alive	7	5	5	3	
Death	2	9	5	2	0.281
Disease-free survival					
No	6	4	4	2	
Yes	1	1	1	1	1
Distant metastasis					
No	10	27	21	7	
Yes	2	3	1	4	**0.016**

Note: Evaluated by Fisher test.
